# Analysis of State-Level Immigrant Policies and Preterm Births by Race/Ethnicity Among Women Born in the US and Women Born Outside the US

**DOI:** 10.1001/jamanetworkopen.2021.4482

**Published:** 2021-04-07

**Authors:** May Sudhinaraset, Rebecca Woofter, Maria-Elena De Trinidad Young, Amanda Landrian, Dovile Vilda, Steven P. Wallace

**Affiliations:** 1 Department of Community Health Sciences, Jonathan and Karin Fielding School of Public Health, University of California, Los Angeles, Los Angeles; 2 Department of Public Health, School of Social Sciences, Humanities, and Arts, University of California, Merced, Merced; 3 Mary Amelia Center for Women’s Health Equity Research, Department of Global Community Health and Behavioral Sciences, Tulane University School of Public Health and Tropical Medicine, New Orleans, Louisiana; 4 Center for Health Policy Research, Jonathan and Karin Fielding School of Public Health, University of California, Los Angeles, Los Angeles

## Abstract

**Importance:**

Criminalizing immigrant policies, a form of structural racism, are associated with preterm birth; however, to date, few population studies have examined this association by race and nativity status or examined the association of inclusive immigrant policies with preterm birth.

**Objective:**

To assess the extent to which variation in preterm birth by race/ethnicity and nativity status is associated with state-level criminalizing vs inclusive immigrant policies.

**Design, Setting, and Participants:**

This retrospective, cross-sectional study analyzed birth record data from all 50 states and the District of Columbia in 2018, as well as state-level indicators of inclusive and criminalizing immigrant policies. White, Black, Asian, and Latina women who had singleton births were included in the study. Statistical analysis was performed from June 1, 2020, to February 5, 2021. Two continuous variables were created to capture the number of criminalizing vs inclusive immigrant policies in effect as of 2017 in each state.

**Main Outcomes and Measures:**

The main outcome measure was preterm birth (<37 weeks’ gestation).

**Results:**

Among the 3 455 514 live births that occurred in 2018, 10.0% were preterm, and 23.2% were to mothers born outside the US. Overall, for women born outside the US, each additional state-level inclusive policy was associated with a 2% decrease in preterm birth (adjusted odds ratio [aOR], 0.98 [95% CI, 0.96-1.00]); there were no significant associations between inclusive policies and preterm birth among women born in the US. In models examining the combined associations of criminalizing and inclusive immigrant policies with preterm birth, each additional criminalizing policy was associated with a 5% increase in preterm birth among Black women born outside the US (aOR, 1.05 [95% CI, 1.00-1.10]). Each additional inclusive immigrant policy was associated with a lower likelihood of preterm birth for Asian women born in the US (aOR, 0.95 [95% CI, 0.93-0.98]) and White women born outside the US (aOR, 0.97 [95% CI, 0.95-0.99]). No significant associations were found among other groups.

**Conclusions and Relevance:**

This study suggests that criminalizing immigrant policies are associated with an increase in preterm birth specifically for Black women born outside the US. Inclusive immigrant policies are associated with a decrease in preterm birth for immigrants overall, Asian women born in the US, and White women born outside the US. No associations were found between criminalizing or inclusive immigrant policies and preterm birth among Latina women.

## Key Points

**Question:**

What is the association of state-level criminalizing immigrant policies vs state-level inclusive immigrant policies with preterm births for immigrants and women of color in the US?

**Findings:**

In this cross-sectional study of 3 455 514 live births in 2018, criminalizing immigrant policies were associated with higher rates of preterm birth for Black women born outside the US, while inclusive immigrant policies were associated with lower preterm birth for all women born outside the US, particularly White women born outside the US.

**Meaning:**

This cross-sectional study suggests that states that have enacted inclusive immigrant policies were associated with higher levels of preterm births among certain populations, even in the context of criminalizing policies.

## Teaser

This cross-sectional study used US birth record data to assess the extent to which variation in preterm birth by race/ethnicity and nativity status is associated with state-level criminalizing or inclusive immigrant policies.

## Introduction

Exclusionary, criminalizing immigrant policies, a form of structural racism, are associated with increased adverse birth outcomes, including preterm birth.^1,2^ These policies criminalize immigrants by barring individuals from certain social protections and rights based on citizenship, impose punitive controls on their presence in the country, and threaten their ability to continue to live in their communities.^3,4^ These policies may contribute to hierarchies of sex, race/ethnicity, and nativity that are associated with inequities in birth outcomes. High rates of preterm birth are a public health priority in the US, with rates for mothers born outside the US increasing steadily since 2014^5^ while decreasing among mothers born in the US.^6^ Although the so-called healthy immigrant effect suggests that immigrants have better birth outcomes compared with women born in the US,^7^ there continues to be a debate about its applicability across immigrant groups, including the need to examine broader structural determinants and racial/ethnic heterogeneity within immigrant groups.^8,9^

Structural racism refers to the historical and contemporary systems that produce and reproduce racial inequities through laws, policies, and practices that are implemented by different levels of government^3,10^ and remain embedded in societal norms.^10^ Policies criminalizing immigrants living in the US, as well as those promoting integration into social and economic institutions, have been enacted mostly at the state or county level during the last 20 years.^11^

Although research is limited, there is growing evidence demonstrating that criminalizing immigrant policies are associated with adverse birth outcomes.^1,2^ Data indicate an increase in preterm birth rates directly following the 2016 presidential election among immigrant and Latina women, suggesting that the acute stress of anti-immigrant rhetoric and changes in federal immigration policies immediately after the election resulted in an increase in preterm birth.^12,13^ Others have found a temporal trend between anti-immigrant rhetoric during pregnancy and subsequent inadequate prenatal care^14^ and low birth weight among Latina women born outside the US.^2^

There are several limitations in the current literature on immigrant policies and birth outcomes. Much of the literature on immigrant policy has focused on exclusionary, criminalizing policies^1,2,12,13^; there is a need to also examine the role of inclusive policies. Although immigrant policies may restrict or extend the rights of noncitizens,^15^ state-level inclusive policies expand immigrant rights and protections, such as access to children’s health insurance regardless of legal status or access to drivers’ licenses.^16^ These types of policies are important to examine because they serve as policy levers to reduce health inequities and may buffer the effects of a more exclusionary national climate and executive actions. In addition, most studies have focused on Latina women.^17,18^ Because of the unique histories and current practices of racialized exclusions across immigrant groups,^19^ it is critical to also examine the experiences of Black and Asian immigrant women. Finally, past studies have examined single policies^2^ or acute events.^1,12,13^ Because individuals experience the impact of an array of policies, the overall policy climate may be a critical marker of health because it captures the dynamics of both exclusion and inclusion. This overall policy climate may be particularly critical for maternal and child health development, for which the cumulative impact of exposures exceeds the impact of a singular policy.^20^

To fill these gaps in the literature, we used national data on all US births in 2018 to examine the associations of criminalizing immigrant policies or inclusive immigrant policies with preterm birth. We also examined how these associations vary across nativity status for Black, Latina, Asian, and White women in the US. We hypothesized that states with higher criminalizing policies would be associated with higher levels of preterm birth, while higher inclusive policies would be associated with lower levels of preterm birth.

## Methods

### Data Source

This retrospective, cross-sectional study includes births from all 50 states and the District of Columbia in 2018. Birth record data were obtained from the National Center for Health Statistics. These data are deidentified and publicly available, and therefore the University of California, Los Angeles determined this study to be exempt from human participants review, and subsequently informed consent was not required. There were a total of 3 801 534 live births in the US in 2018. We excluded women with nonsingleton births, women who did not identify with one of our main race/ethnicity categories, and women who had missing covariates and outcome data (eTable 1 in the Supplement). In total, 346 020 women were excluded, for a final analytic sample of 3 455 514 live births. The primary outcome of interest was preterm birth, defined as birth before 37 completed weeks of gestation. This study followed the Strengthening the Reporting of Observational Studies in Epidemiology (STROBE) reporting guideline.

### Independent Variables

Two continuous variables were created to capture the number of criminalizing and inclusive immigrant policies in effect as of December 31, 2017, in each state, referring to the preconception year prior to when women gave birth (Figure). The selected policies have been previously identified and described in detail elsewhere (eTable 2 in the Supplement).^16^ We conducted a policy scan of 6 criminalizing immigrant policies, categorized as such because they create mechanisms of surveillance and immigration enforcement across the following 3 sectors: work authorization, immigration enforcement and criminal justice, and identification and licensing. Next, we conducted a scan of 14 inclusive immigrant policies spanning the following 4 sectors: health and social service benefits, education, labor and employment, and language access. These policies were categorized as such because they grant noncitizen residents access to state institutions regardless of citizenship or legal status. States were given a 1 if the policy was in effect and a 0 if it was not. These values were then summed to create continuous criminalizing and inclusive immigrant policy indices for each state; index scores ranged from 0 to 6 for the criminalizing immigrant policy variable (whereby an increasing score denoted a more criminalizing immigrant policy context) and from 0 to 14 for the inclusive immigrant policy variable (whereby an increasing score denoted a more inclusive immigrant policy context).

**Figure.**
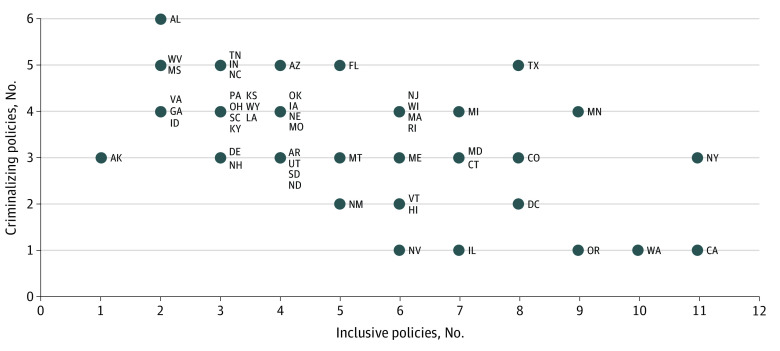
Criminalizing and Inclusive Immigrant Policies by State

### Covariates

Individual-level covariates were obtained from birth records and included self-reported maternal race/ethnicity (non-Hispanic White, non-Hispanic Black, non-Hispanic Asian, and Hispanic), which was classified in accordance with the guidelines from the Office of Management and Budget^21^; nativity (born outside the US vs born inside the US); age (categorized as <20, 20-24, 25-29, 30-34, 35-39, and ≥40 years); educational level (<high school; high school graduate or GED [General Educational Development Certification]; some college, associate’s degree, or bachelor’s degree; and graduate or higher degree); smoked at any stage during pregnancy (yes or no); and insurance type (public [Medicaid], private, and self-pay or other). For the remainder of the article, non-Hispanic White women are referred to as “White,” non-Hispanic Black women are referred to as “Black,” and Hispanic women are referred to as “Latina.” State-level covariates include the percentage of each state’s residents born outside of the US in 2017,^22^ percentage living below the federal poverty level in 2017,^23^ and percentage of voters in the 2016 presidential election voting for the Republican presidential candidate.^24^

### Statistical Analysis

Statistical analysis was performed from June 1, 2020, to February 5, 2021. We calculated descriptive statistics across individual-level and state-level variables, including the percentage of preterm births across each variable. Mixed-effects multilevel logistic regression models were used to estimate the odds of preterm birth associated with a 1-unit increase in criminalizing and inclusive policies. An exploratory analysis of the policy indices included examining the distribution of preterm births by each policy level as well as plotting the estimated probabilities by preterm birth by increasing level of policy. Because there was a linear association between the policy indices and preterm birth, the policy indices were modeled as continuous variables (eFigure 1 and eFigure 2 in the Supplement). Based on a priori conceptualization of differences in policy effects by race/ethnicity and nativity status, models were stratified by race/ethnicity and nativity status. First, we ran models for preterm birth and criminalizing policies and inclusive policies, with each policy modeled independently, adjusted for all individual-level and state-level covariates (eTable 3 and eTable 4 in the Supplement). Second, we ran a model for preterm birth with both criminalizing and inclusive immigrant policies in the model, adjusting for all individual-level and state-level covariates. We tested models using an interaction term between inclusive and criminalizing policies; however, interaction terms for the total population were not significant, and the 95% CIs by nativity status overlapped with 1.0 (eTable 5 and eTable 6 in the Supplement); we therefore modeled the policy indices separately. Finally, we ran these models stratified by nativity and race/ethnicity. We finalized models by testing for collinearity using the variance inflation factor and goodness-of-fit tests. In addition, we conducted sensitivity analyses using 2015 immigrant policy indices with 2018 data to examine whether there was a time-lagged association between policy and birth outcomes (eTable 7, eTable 8, and eTable 9 in the Supplement). We also ran sensitivity models to exclude the policy indicator of coverage of prenatal care regardless of documentation status from the inclusive policy index because this policy may be particularly salient to birth outcomes (eTable 10, eTable 11, and eTable 12 in the Supplement). Because the results were substantially the same and our hypothesis focuses on stress during pregnancy, we present only the 2018 findings. Statistical significance was assessed at a 2-sided *P* < .05. All analyses were conducted using Stata, version 16 MP software (StataCorp LLC).^25^

## Results

### Study Sample

Of the 3 455 514 live births in the analysis, 10.0% were preterm, and 23.2% were to mothers born outside the US (Table 1). Black women had the highest preterm birth rate at 15.1%, followed by 10.4% of Latina women, 8.5% of White women, and 8.4% of Asian women. Women born in the US had higher levels of preterm birth than women born outside the US (10.1% vs 9.6%).

**Table 1.** Characteristics of Births and Frequency of Preterm Birth, 2018^a^

Characteristic	No. (%)	
	Total in population	Preterm birth within each category
Preterm birth	344 017 (10.0)	NA
Maternal race/ethnicity		
Asian	229 012 (6.6)	19 264 (8.4)
Black	517 497 (15.0)	78 279 (15.1)
Latina	852 180 (24.7)	88 509 (10.4)
White	1 856 825 (53.7)	157 965 (8.5)
Maternal nativity		
US	2 653 654 (76.8)	266 898 (10.1)
Outside the US	801 860 (23.2)	77 119 (9.6)
Maternal age, y		
<20	164 588 (4.8)	20 926 (12.7)
20-24	661 538 (19.1)	68 467 (10.4)
25-29	1 006 057 (29.1)	92 740 (9.2)
30-34	998 101 (28.9)	90 456 (9.1)
35-39	512 603 (14.8)	55 572 (10.8)
≥40	112 627 (3.3)	15 856 (14.1)
Maternal educational level		
Less than high school	437 995 (12.7)	57 699 (13.2)
High school graduate or GED	888 549 (25.7)	102 381 (11.5)
Some college, associate’s degree, or bachelor’s degree	1 699 026 (49.2)	152 993 (9.0)
Graduate degree or higher	429 944 (12.4)	30 944 (7.2)
Insurance status		
Private	1 724 368 (49.9)	142 423 (8.3)
Public	1 514 028 (43.8)	180 126 (11.9)
Self-pay or other	217 118 (6.3)	21 468 (9.9)
Smoked during pregnancy		
No	3 236 273 (93.7)	311 858 (9.6)
Yes	219 241 (6.3)	32 159 (14.7)
State-level characteristics, mean (SD), %		
Born outside the US	9.5 (6.2)	NA
Below federal poverty level	12.3 (2.9)	NA
Voted Republican in 2016 presidential election	48.3 (12.0)	NA

Abbreviations: GED, General Educational Development Certification; NA, not applicable.

^a^ The final analytic sample consisted of 3 455 514 live births in 2018.

### Criminalizing and Inclusive Policies by Nativity Status

In multivariable models with the full analytic sample, neither criminalizing nor inclusive policies were significantly associated with increased odds of preterm birth (criminalizing: adjusted odds ratio [aOR], 1.03 [95% CI, 1.00-1.05]; inclusive: aOR, 0.99 [95% CI, 0.98-1.01]) (Table 2). We then assessed the associations of criminalizing policies or inclusive policies with preterm birth by nativity status (Table 3). Overall, for women born outside the US, each additional inclusive policy in their state was associated with a 2% decrease in preterm birth (aOR, 0.98 [95% CI, 0.96-1.00]); there were no significant associations between inclusive policies and preterm birth among women born in the US (aOR, 1.00 [95% CI, 0.98-1.01]). Overall, there were no significant associations between criminalizing policies and preterm birth for either women born in the US or women born outside the US, controlling for covariates and inclusive policy.

**Table 2.** Multilevel Logistic Models Estimating Odds of Preterm Birth^a^

Characteristic	aOR (95% CI)	*P* value
State immigrant policy context		
Criminalizing policy	1.03 (1.00-1.05)	.07
Inclusive policy	0.99 (0.98-1.01)	.47
Individual-level variables		
Maternal race/ethnicity		
White	1 [Reference]	NA
Asian	1.30 (1.28-1.32)	<.001
Black	1.73 (1.72-1.75)	<.001
Latina	1.30 (1.28-1.31)	<.001
Maternal nativity		
US	1 [Reference]	NA
Outside the US	0.85 (0.84-0.86)	<.001
Maternal age, y		
<20	1 [Reference]	NA
20-24	0.91 (0.89-0.93)	<.001
25-29	0.93 (0.91-0.94)	<.001
30-34	1.05 (1.03-1.06)	<.001
35-39	1.31 (1.29-1.33)	<.001
≥40	1.74 (1.70-1.78)	<.001
Maternal educational level		
Less than high school	1 [Reference]	NA
High school or GED	0.88 (0.87-0.89)	<.001
Some college, associate’s degree, or bachelor’s degree	0.74 (0.74-0.75)	<.001
Graduate degree or higher	0.62 (0.61-0.63)	<.001
Insurance status		
Private	1 [Reference]	NA
Public	1.18 (1.17-1.19)	<.001
Self-pay	1.03 (1.02-1.05)	<.001
Smoked during pregnancy		
No	1 [Reference]	NA
Yes	1.47 (1.45-1.49)	<.001
State-level characteristics		
% Born outside the US	1.00 (0.99-1.00)	.69
% Below FPL	1.02 (1.01-1.03)	<.001
% Voted Republican in 2016 presidential election	1.00 (1.00-1.01)	.12

Abbreviations: aOR, adjusted odds ratio; FPL, federal poverty level; GED, General Educational Development Certification; NA, not applicable.

^a^ The final analytic sample consisted of 3 455 514 live births in 2018.

**Table 3.** Mixed-Effects Logistic Models Estimating Odds of Preterm Birth and State Immigrant Policies, Stratified by Nativity of Mother^a^

Nativity of mother	No.	Criminalizing policy (range, 0-6)		Inclusive policy (range, 0-14)	
		aOR (95% CI)	*P* value	aOR (95% CI)	*P* value
US	2 653 654	1.02 (1.00-1.05)	.08	1.00 (0.98-1.01)	.87
Outside the US	801 860	1.02 (0.99-1.05)	.29	0.98 (0.96-1.00)	.03

Abbreviation: aOR, adjusted odds ratio.

^a^ Models adjusted for individual-level variables: maternal race/ethnicity, age, educational level, insurance status, and smoking status during pregnancy; and state-level variables: percentage Republican voters in 2016 presidential election and percentage living below the federal poverty level; models are adjusted for both policies.

### Criminalizing and Inclusive Policies by Race/Ethnicity and Nativity Status

In models examining the combined associations of criminalizing and inclusive policies with preterm birth, each additional state-level criminalizing policy was associated with a 5% increase in preterm birth among Black women born outside the US (aOR, 1.05 [95% CI, 1.00-1.10]) (Table 4). The association between inclusive policies and preterm birth was significant for Asian women born in the US (aOR, 0.95 [95% CI, 0.93-0.98]) and White women born outside the US (aOR, 0.97 [95% CI, 0.95-0.99]). Although each additional inclusive policy was associated with a 3% decrease in preterm birth among Black women born outside the US when not controlling for criminalizing policies (eTable 4 in the Supplement), once criminalizing policies were accounted for, inclusive policies were no longer associated with a reduction in preterm birth for Black women born outside the US. No significant associations were found among other groups.

**Table 4.** Mixed-Effects Logistic Models Estimating Odds of Preterm Birth and State Immigrant Policies, Stratified by Race/Ethnicity and Nativity of Mother^a^

Race/ethnicity and nativity of mother	No.	Criminalizing policy (range, 0-6)		Inclusive policy (range, 0-14)	
		aOR (95% CI)	*P* value	aOR (95% CI)	*P* value
Asian (n = 229 012)					
US	43 536	0.95 (0.91-1.00)	.06	0.95 (0.93-0.98)	<.001
Outside the US	185 476	1.00 (0.95-1.04)	.83	0.98 (0.96-1.01)	.14
Black (n = 517 497)					
US	429 754	1.01 (0.98-1.05)	.45	1.00 (0.98-1.02)	.96
Outside the US	87 743	1.05 (1.00-1.10)	.03	0.98 (0.95-1.00)	.08
Latina (n = 852 180)					
US	448 854	1.02 (0.99-1.05)	.30	1.00 (0.98-1.02)	.95
Outside the US	403 326	1.02 (0.99-1.06)	.18	0.99 (0.97-1.01)	.23
White (n= 1 856 825)					
US	731 510	1.02 (0.99-1.05)	.15	1.00 (0.98-1.01)	.78
Outside the US	125 315	0.99 (0.95-1.03)	.68	0.97 (0.95-0.99)	.02

Abbreviation: aOR, adjusted odds ratio.

^a^ Models adjusted for individual-level variables: maternal age, educational level, insurance status, and smoking status during pregnancy; and state-level variables: percentage born outside the US, percentage Republican voters in 2016 presidential election, and percentage living below the federal poverty level; models are adjusted for both policies.

## Discussion

This national study found that Black women born outside the US who lived in states with more criminalizing immigrant policies had higher odds of preterm birth. The study also found that, overall, immigrant women living in states with more inclusive policies were less likely to have preterm births, even in the context of criminalizing policies. However, there were significant racial/ethnic inequities in the protective associations of inclusive policies with preterm birth.

In particular, criminalizing policies were associated with a 5% higher odds of preterm birth for Black women born outside the US, even in the context of inclusive policies. This finding indicates that inclusive policies may not counter the negative association of criminalizing policies for Black women born outside the US. These findings may reflect a “double jeopardy” for Black women born outside the US who experience racial profiling and exclusion across both the criminal justice and immigration systems. This finding is consistent with other evidence that Black immigrants, despite being only 7% of the US population who were born outside the US,^26^ disproportionately experience immigration enforcement. For example, despite being a population that is largely documented, Black immigrants with lawful permanent status are disproportionately deported based on criminal grounds.^26^ Thus, the presence of more criminalizing policies may increase fear and stress among Black women born outside the US who are concerned about the vulnerability of their legal status (or that of their partners, family, or friends) and about the risk of entering the “prison-to-deportation pipeline” amid an already racist, punitive criminal justice system.^27^

In addition, these findings suggest increased experiences of discrimination or “racialized othering” among Black immigrant women at the intersections of sex, race/ethnicity, and immigration status, which may be more common in states with criminalizing immigrant policies.^28^ Most Black immigrants come from Caribbean or African countries where they are the racial majority,^29^ and thus they may be facing this racialization for the first time. Alternatively, these findings may reflect a “double invisibility” of Black immigrants, particularly in contexts with more criminalizing policies, whereby their minority racial/ethnic status and nativity status render their distinct needs unacknowledged and unaddressed in broader social and political institutions.^28^ The added psychological and economic distress may be associated with the short-term and long-term health of Black immigrant women before and during pregnancy.

Exclusionary state-level immigrant policies may operate through multiple complex pathways to produce inequities in birth outcomes,^3,10^ including increasing maternal stress in utero.^30,31^ Although it remains unclear in what contexts and which particular subgroups of women are particularly susceptible to stress-induced physiological responses,^32^ a substantial body of literature points to the assocations between the important role of context, stress during pregnancy, and increased preterm birth risks.^32,33,34^ More proximal pathways linking policies to birth outcomes may include restricting access to health-promoting resources, such as prenatal care,^35^ public assistance, or follow-up visits,^36^ and impairing social capital and trust in public institutions.^37^

This study also found that inclusive policies were associated with a lower odds of preterm birth among immigrants overall, Asian women born in the US, and White women born outside the US. Asian women born in the US, but not Asian women born outside the US, were protected by inclusive policies. This outcome may be the result of “spillover effects,” or the impact of exclusionary policies and anti-immigrant rhetoric on the broader community regardless of their own legal status.^38^ In the US, 16.7 million people nationwide live in a mixed-status family (ie, families that include undocumented individuals),^39^ with 1.7 million undocumented Asian individuals living in the US.^40^ Moreover, knowing someone who has been deported, regardless of legal status, is associated with adverse health consequences.^41^ This study suggests that there may also be potential spillover effects of inclusive policies on Asian women. Further examination to unpack the heterogeneity of Asian people born in the US is warranted based on these findings.

In addition, this study found no significant associations for Latina women across nativity status. One hypothesis for this finding is that most of the anti-immigrant rhetoric has focused over time on Latinos,^42^ with periodic anti-Muslim and anti-Chinese rhetoric, resulting in high levels of exposure to xenophobia and discrimination that may not vary significantly across states. It is possible, therefore, that the overarching national context surpasses state-level actions, resulting in less importance of state variation for Latinas. Moreover, other factors may be at play, including immigrant legal status and length of time in the US.

This study has several research and public health implications. Given the increasing recognition of the critical role that structural racism plays in maternal health and birth outcomes, more research is needed to examine how criminalizing immigrant policies may harm the health of immigrants and how inclusive policies may buffer some of those harmful effects. Future research should examine how social policies are associated with immigrant reproductive health outcomes. Furthermore, there is a need to examine the association between immigrant policies and birth outcomes through an intersectional lens—one that recognizes the multiple social identities, processes, and statuses that may lead to experiences of exclusion.^17^ Such research is needed to inform and develop policy measures that are more reflective of immigrant women’s experiences of exclusion and inclusion across the life span. Finally, policy makers can address birth inequities through state-level and national-level policies that promote immigrant integration.

### Limitations and Strengths

This study has some limitations. First, we were unable to identify documentation status or time in the US from the National Center for Health Statistics data used. It is expected that those who are undocumented or lawful permanent residents (“green card” holders) and those who have lived in the US for longer periods would be the most impacted by both criminalizing and inclusive policies. However, past studies have reported mixed results when assessing birth outcomes across mothers’ documentation status.^43,44^

Furthermore, this study did not include Pacific Islanders, nor did it assess heterogeneity across subgroups of Latina and non-Latina Black and Asian women. Previous studies have demonstrated that women born in sub-Saharan African countries have lower rates of preterm birth and small-for-gestational age offspring compared with Black women born in the Caribbean.^45^ Another study found marked heterogeneity in birth outcomes comparing women born in the US with those born outside the US across numerous Asian subgroups.^46^ Moreover, in birth certificate data, “race” and “Hispanic ethnicity” are recorded as separate items; therefore, someone can identify as Hispanic ethnicity as well as White, Black, or other racial categories. However, evidence shows high validity between birth record data for race/ethnicity categories and self-reported identification.^47^

Although this study accounted for a number of individual and state-level covariates, there may be unmeasured confounding and the possibility of findings being due to chance as a result of the large number of comparisons. This study also included cross-sectional data and is therefore unable to examine the causal effects of immigrant policies. Future studies examining dates of specific policy enactment could provide rigorous evidence on the association of immigrant policies with adverse birth outcomes.

Despite these limitations, this study has several strengths. First, to our knowledge, there are few empirical studies that aim to quantify the association of structural racism with population health. These findings highlight the importance of interrogating inequities in birth outcomes through the lens of structural racism and how inclusive policies can counteract restrictive policies. Second, this study examines the overall policy context as opposed to a single policy. Third, this study uses national data to examine not only the experiences of Latina women but also other immigrant groups, including Black and Asian women.

## Conclusions

Criminalizing immigrant policies are associated with an increase in preterm birth specifically for Black women born outside the US. Inclusive immigrant policies are associated with a decrease in preterm birth for immigrants overall, with heterogeneity across race/ethnicity and nativity. Sociopolitical factors continue to be associated with racial/ethnic disparities in preterm birth for women born in the US or outside the US.

## Notes

Supplement. **eTable 1.** Missing and Excluded Data by Variable **eTable 2.** List of Policy Indicators by Sector **eTable 3.** Mixed Effects Multivariable Logistic Models Estimating Odds of Preterm Birth and State Immigrant Policies, Stratified by Nativity of Mother **eTable 4.** Mixed Effects Logistic Multivariable Models Estimating Odds of Preterm Birth and State Immigrant Policies, Stratified by Race and Nativity of Mother **eTable 5.** Multilevel Logistic Models Estimating Odds of Preterm Birth, With an Interaction Between Policy Variables (N=3,455,514) **eTable 6.** Mixed Effects Logistic Models Estimating Odds of Preterm Birth and State Immigrant Policies and Interaction Between Policy Variables, Stratified by Nativity of Mother **eTable 7.** Multilevel Logistic Models Estimating Odds of Preterm Birth and 2015 State Immigrant Policies, 2018 Birth Data (N=3,455,514) **eTable 8.** Mixed Effects Logistic Models Estimating Odds of Preterm Birth and 2015 Immigrant Policies, Stratified by Nativity of Mother **eTable 9.** Mixed Effects Logistic Models Estimating Odds of Preterm Birth and 2015 State Immigrant Policies, Stratified by Race and Nativity of Mother **eTable 10.** Multilevel Logistic Models Estimating Odds of Preterm Birth, With Prenatal Care Removed From Inclusive Policy Index (N=3,455,514) **eTable 11.** Mixed Effects Logistic Models Estimating Odds of Preterm Birth From Criminalizing and Inclusive Policy Variables, With Prenatal Care Removed From Inclusive Policy Index, Stratified by Nativity of Mother **eTable 12.** Mixed Effects Logistic Models Estimating Odds of Preterm Birth From Criminalizing and Inclusive Policy Variables, With Prenatal Care Removed From Inclusive Policy Index, Stratified by Race and Nativity of Mother **eFigure 1.** Predictive Margins With 95% CI, Criminalizing Policies **eFigure 2.** Predictive Margins With 95% CI, Inclusive Policies
