# Supplementary material for: Analysis of State-Level Immigrant Policies and Preterm Births by Race/Ethnicity Among Women Born in the US and Women Born Outside the US
Source: JAMA Netw Open. 2021 Apr 7;4(4):e214482. doi: 10.1001/jamanetworkopen.2021.4482 (PMC8027912; doi:10.1001/jamanetworkopen.2021.4482)
Supplement: Supplement. — eTable 1. Missing and Excluded Data by Variable eTable 2. List of Policy Indicators by Sector eTable 3. Mixed Effects Multivariable Logistic Models Estimating Odds of Preterm Birth and State Immigrant Policies, Stratified by Nativity of Mother eTable 4. Mixed Effects Logistic Multivariable Models Estimating Odds of Preterm Birth and State Immigrant Policies, Stratified by Race and Nativity of Mother eTable 5. Multilevel Logistic Models Estimating Odds of Preterm Birth, With an Interaction Between Policy Variables (N=3,455,514) eTable 6. Mixed Effects Logistic Models Estimating Odds of Preterm Birth and State Immigrant Policies and Interaction Between Policy Variables, Stratified by Nativity of Mother eTable 7. Multilevel Logistic Models Estimating Odds of Preterm Birth and 2015 State Immigrant Policies, 2018 Birth Data (N=3,455,514) eTable 8. Mixed Effects Logistic Models Estimating Odds of Preterm Birth and 2015 Immigrant Policies, Stratified by Nativity of Mother eTable 9. Mixed Effects Logistic Models Estimating Odds of Preterm Birth and 2015 State Immigrant Policies, Stratified by Race and Nativity of Mother eTable 10. Multilevel Logistic Models Estimating Odds of Preterm Birth, With Prenatal Care Removed From Inclusive Policy Index (N=3,455,514) eTable 11. Mixed Effects Logistic Models Estimating Odds of Preterm Birth From Criminalizing and Inclusive Policy Variables, With Prenatal Care Removed From Inclusive Policy Index, Stratified by Nativity of Mother eTable 12. Mixed Effects Logistic Models Estimating Odds of Preterm Birth From Criminalizing and Inclusive Policy Variables, With Prenatal Care Removed From Inclusive Policy Index, Stratified by Race and Nativity of Mother eFigure 1. Predictive Margins With 95% CI, Criminalizing Policies eFigure 2. Predictive Margins With 95% CI, Inclusive Policies [file jamanetwopen-e214482-s001.pdf]

Supplementary Online Content

Sudhinaraset M, Woofter R, Young MEDT, Landrian A, Vilda D, Wallace SP. Analysis of state-level immigrant policies and preterm births by race/ethnicity among women born in the US and women born outside the US. *JAMA Netw Open.* 2021;4(4):e214482. doi:10.1001/jamanetworkopen.2021.4482

eTable 1. Missing and Excluded Data by Variable
eTable 2. List of Policy Indicators by Sector
eTable 3. Mixed Effects Multivariable Logistic Models Estimating Odds of Preterm Birth and State Immigrant Policies, Stratified by Nativity of Mother
eTable 4. Mixed Effects Logistic Multivariable Models Estimating Odds of Preterm Birth and State Immigrant Policies, Stratified by Race and Nativity of Mother
eTable 5. Multilevel Logistic Models Estimating Odds of Preterm Birth, With an Interaction Between Policy Variables (N=3,455,514)
eTable 6. Mixed Effects Logistic Models Estimating Odds of Preterm Birth and State Immigrant Policies and Interaction Between Policy Variables, Stratified by Nativity of Mother
eTable 7. Multilevel Logistic Models Estimating Odds of Preterm Birth and 2015 State Immigrant Policies, 2018 Birth Data (N=3,455,514)
eTable 8. Mixed Effects Logistic Models Estimating Odds of Preterm Birth and 2015 Immigrant Policies, Stratified by Nativity of Mother
eTable 9. Mixed Effects Logistic Models Estimating Odds of Preterm Birth and 2015 State Immigrant Policies, Stratified by Race and Nativity of Mother
eTable 10. Multilevel Logistic Models Estimating Odds of Preterm Birth, With Prenatal Care Removed From Inclusive Policy Index (N=3,455,514)
eTable 11. Mixed Effects Logistic Models Estimating Odds of Preterm Birth From Criminalizing and Inclusive Policy Variables, With Prenatal Care Removed From Inclusive Policy Index, Stratified by Nativity of Mother
eTable 12. Mixed Effects Logistic Models Estimating Odds of Preterm Birth From Criminalizing and Inclusive Policy Variables, With Prenatal Care Removed From Inclusive Policy Index, Stratified by Race and Nativity of Mother
eFigure 1. Predictive Margins with 95% CI, Criminalizing Policies
eFigure 2. Predictive Margins with 95% CI, Inclusive Policies

This supplementary material has been provided by the authors to give readers additional information about their work.

© 2021 Sudhinaraset M et al. *JAMA Network Open.*

eTable 1. Missing and Excluded Data by Variable

**Variable**	**N (%) Missing**
Race	32,441 (0.85)
Nativity	6,183 (0.16)
Education	28,189 (0.74)
Delivery Payment Type	20,041 (0.53)
Smoking	13,123 (0.35)
Low birthweight	1,822 (0.05)
Preterm birth	1,287 (0.03)
**Subgroups Excluded**	**N (%) Removed**
Plural pregnancies	127,419 (3.35)
American Indian/Alaskan Native	27,478 (0.72)
Native Hawaiian/Other Pacific Islander	8,689 (0.23)
Multiracial	79,348 (2.09)

© 2021 Sudhinaraset M et al. *JAMA Network Open.*

eTable 2. List of Policy Indicators by Sector

CRIMINALIZATION POLICIES		
**Sector**	**Policy**	**Indicator that policy exists (Yes=1/No=0)**
**Identification and licensing**	State driver's licenses	Does the state require a social security number to obtain a driver's license?[a]
	Compliance with the federal Real ID Act of 2005 which sets standards for state licenses/IDs	Does the state comply with REAL ID?[b]
**Work authorization**	Use of employment authorization database, E-Verify	Does the state mandate employers use E-Verify?[c]
**Immigration enforcement and criminal justice**	Law enforcement collaboration with federal enforcement	Does the state fully collaborate with federal immigration authorities[d]
	Law enforcement inquiry about legal status	Does the state require or allow that law enforcement verify individuals' legal status at the time of a stop or arrest?[e]
	Sentencing laws	Does the state sentence non-violent criminal offenses at least 365 days?[e]
**INTEGRATION POLICIES**		
**Sector**	**Policy**	**Indicator that policy exists (Yes=1/No=0)**
**Health and social service benefits**	State Children's Health Insurance Program (SCHIP)	Does state provide health insurance to children regardless of legal status?[f]
	Medicaid - Prenatal care	Does state provide care to pregnant women regardless of legal status?[f]
	Supplemental Nutrition Assistance Program	Does state count a prorated share of ineligible non-citizen income to determine family eligibility for benefits?[g]
**Education**	In-state college and university tuition	Does the state provide most students in-state tuition regardless of legal status?[h]
	Financial aid for colleges and universities	Does the state provide students scholarships or financial aid regardless of legal status?[h]
**Labor and Employment**	Citizenship requirements for peace officers	Does the state require peace officers be citizens?[i]
	Citizenship requirements for teachers	Does the state require teachers be citizens?[j]
	Worker's compensation	Does state include undocumented immigrants in the definition of employee?[k]
	Extension of protections for agricultural workers	Does the state extend wage and hour protections for agricultural workers?[l]
	Extension of protections for domestic workers	Does the state extend wage and hour protections for domestic workers?[l]
	Domestic Worker's Bill of Rights	Does the state have a Domestic Worker's Bill of Rights?[l]

© 2021 Sudhinaraset M et al. *JAMA Network Open.*

	Protection against immigration-related employer retaliation	Does the state have laws that protect noncitizen workers from employer retaliation related to their legal status?[m]
**Language access**	Payment of interpreters through Medicaid and/or SCHIP	Does the state pay for interpreters through Medicaid or SCHIP?[n]
	English language-only legislation	Does the state have English as the official language?[o]
**Sources**		
[a] National Council of State Legislatures, Immigrant Policy Project. Available at http://www.ncsl.org/research/immigration/states-offering-driver-s-licenses-to-immigrants.aspx		
[b] National Council of State Legislatures. Available at: http://www.ncsl.org/documents/standcomm/sctran/REALIDComplianceReport.pdf		
[c] National Council of State Legislatures, State E-Verify Action. Available at http://www.ncsl.org/research/immigration/everify-faq.aspx		
[d] National Council of State Legislatures. Available at: https://www.ilrc.org/sites/default/files/resources/rise_of_sanctuary-lg-20180201.pdf		
[e] César Cuauhtémoc García Hernández. *Crimmigration Law.* American Bar Association. 2015 and author's review of state statutes.		
[f] Health Care Coverage Maps, National Immigration Law Center, Available at: https://www.nilc.org/issues/health-care/healthcoveragemaps/		
[g] Supplemental Nutrition Assistance Program State Options Report, United States Department of Agriculture, Food and Nutrition Service, 14th Edition (May 2018), Available at: https://fns-prod.azureedge.net/sites/default/files/snap/14-State-Options.pdf		
[h] Undocumented Student Tuition: State Action, National Council of State Legislatures, Available at: http://www.ncsl.org/research/education/undocumented-student-tuition-state-action.aspx#2		
[i] Author's review of state law enforcement agency hiring requirements and legislative codes		
[j] Author's review of state department of education hiring requirements and legislative codes		
k "Working in the Shadows: Illegal Aliens' Entitlement to State Workers' Compensation," (2004) Schumann J, 89 Iowa Law Review pp 709-739 and author's review of state statutes		
[l] National Employment Law Project, Winning Wage Justice: An Advocate's Guide to State and City Policies to Fight Wage Theft: Available at: http://www.nelp.org/content/uploads/2015/03/WinningWageJustice2011.pdf		
[m] National Immigration Law Center, Immigrant-inclusive State and Local Policies Move Ahead in 2014-15: Available at: https://www.nilc.org/wp-content/uploads/2016/02/pro-immigrant-policies-move-ahead-2015-12.pdf		
[n] Mara Youdelman, National Health Law Program, Medicaid and CHIP Reimbursement Models for Language Services: Available at: https://healthlaw.org/resource/medicaid-and-chip-reimbursement-models-for-language-services/		
[o] US English, State Legislation: Available at: https://www.usenglish.org/legislation/state/		

© 2021 Sudhinaraset M et al. *JAMA Network Open.*

eTable 3. Mixed Effects Multivariable Logistic Models Estimating Odds of Preterm Birth and State Immigrant Policies, Stratified by Nativity of Mother

	**Model 1**		**Model 2**	
	**Criminalizing Policy (range 0-6)**		**Inclusive Policy (range 0-14) P-value**	
	**AOR (95% CI)**	***P* value**	**AOR (95% CI)**	***P* value**
**US-born (N=2,653,654)**	1.02 (1.00, 1.05)	0.06	0.99 (0.98, 1.01)	0.45
**Foreign-born (N=801,860)**	1.03 (1.00, 1.06)	0.07	0.98 (0.96, 0.99)	0.008

Models adjusted for individual-level variables: maternal race, age, education, delivery payment type, and smoking status during pregnancy, and state level variables: state-level percent Republican voters in 2016 presidential election, and state-level percent living below the Federal Poverty Line

© 2021 Sudhinaraset M et al. *JAMA Network Open.*

eTable 4. Mixed Effects Logistic Multivariable Models Estimating Odds of Preterm Birth and State Immigrant Policies, Stratified by Race and Nativity of Mother

	**Model 1**		**Model 2**	
	**Criminalizing Policy (range 0-6)**		**Inclusive Policy (range 0-14)**	
	**AOR (95% CI)**	***P* value**	**AOR (95% CI)**	***P* value**
**Asian (N=229,012)**				
US-born Asian (N=43,536)	0.98 (0.92, 1.04)	0.43	0.96 (0.93, 0.99)	0.003
Foreign-born Asian (N=185,476)	1.00 (0.96, 1.05)	0.82	0.98 (0.96, 1.01)	0.13
**Black (N=517,497)**				
US-born Black (N=429,754)	1.01 (0.98, 1.05)	0.42	1.00 (0.98, 1.02)	0.76
Foreign-born Black (N=87,743)	1.07 (1.02, 1.12)	0.005	0.97 (0.94, 0.99)	0.01
**Latina (N=852,180)**				
US-born Latina (N=448,854)	1.02 (0.99, 1.05)	0.28	1.00 (0.98, 1.02)	0.77
Foreign-born Latina (N=403,326)	1.03 (1.00, 1.07)	0.06	0.98 (0.96, 1.00)	0.07
**White (N=1,856,825)**				
US-born White (N=731,510)	1.02 (1.00, 1.05)	0.11	0.99 (0.98, 1.01)	0.43
Foreign-born White (N=125,315)	1.01 (0.97, 1.05)	0.67	0.97 (0.95, 0.99)	0.02

Models adjusted for individual level variables: maternal age, education, delivery payment type, and smoking status during pregnancy, and state-level variables: state-level percent foreign, state-level percent Republican voters in 2016 presidential election, and state-level percent living below the Federal Poverty Line

eTable 5. Multilevel Logistic Models Estimating Odds of Preterm Birth, With an Interaction Between Policy Variables (N=3,455,514)

	**AOR (95% CI)**	***P* value**
State Immigrant Policy Context		
**Criminalizing Policy**	0.99 (0.94, 1.05)	0.78
**Inclusive Policy**	0.98 (0.95, 1.01)	0.14
**Criminalizing*Inclusive Policy**	1.01 (1.00, 1.01)	0.20
Individual-level variables		
**Maternal Race**		
White	Ref	
Asian	1.30 (1.28, 1.32)	0.000
Black	1.73 (1.72, 1.75)	0.000
Hispanic	1.30 (1.28, 1.31)	0.000
**Maternal Nativity**		
US born	Ref	
Foreign born	0.85 (0.84, 0.86)	0.000
**Maternal Age**		
<=19	Ref	
20-24	0.91 (0.89, 0.93)	0.000
25-29	0.93 (0.91, 0.94)	0.000
30-34	1.05 (1.03, 1.06)	0.000
35-39	1.31 (1.29, 1.33)	0.000
40+	1.74 (1.70, 1.78)	0.000
**Maternal Education**		
Less than HS	Ref	
HS	0.88 (0.87, 0.89)	0.000
Some College/BA	0.74 (0.74, 0.75)	0.000
Graduate School	0.62 (0.61, 0.63)	0.000
**Delivery Payment**		
Private	Ref	
Public	1.18 (1.17, 1.19)	0.000
Self-Pay	1.03 (1.02, 1.05)	0.000
**Smoked in Pregnancy**		
No	Ref	
Yes	1.47 (1.45, 1.49)	0.000
**State-level Percent Foreign-Born**	1.00 (0.99, 1.00)	0.74
**State-level Percent Below FPL**	1.02 (1.01, 1.03)	0.000
**State-level Percent Republican**	1.00 (1.00, 1.01)	0.09

© 2021 Sudhinaraset M et al. *JAMA Network Open.*

eTable 6. Mixed Effects Logistic Models Estimating Odds of Preterm Birth and State Immigrant Policies and Interaction Between Policy Variables, Stratified by Nativity of Mother

**State Immigrant Policy Context**	**Criminalizing Policy (range 0-6)**	**Inclusive Policy (range 0-14)**	**Criminalizing*Inclusive Policy**
	**AOR (95% CI)**	**AOR (95% CI)**	**AOR (95% CI)**
**US-born (N=2,653,654)**	0.99 (0.94, 1.05)	0.98 (0.95, 1.01)	1.01 (1.00, 1.01)
**Foreign-born (N=801,860)**	0.95 (0.89, 1.02)	0.95 (0.92, 0.98)**	1.01 (1.00, 1.02)*

*p<0.05, **p<0.01, **p<0.001
Models adjusted for individual-level variables: maternal race, age, education, delivery payment type, and smoking status during pregnancy, and state level variables: state-level percent Republican voters in 2016 presidential election, and state-level percent living below the Federal Poverty Line

© 2021 Sudhinaraset M et al. *JAMA Network Open.*

eTable 7. Multilevel Logistic Models Estimating Odds of Preterm Birth and 2015 State Immigrant Policies, 2018 Birth Data (N=3,455,514)

	**AOR (95% CI)**	***P* value**
State Immigrant Policy Context		
**Criminalizing Policy**	1.03 (1.00, 1.05)	0.07
**Inclusive Policy**	0.99 (0.98, 1.01)	0.47
Individual-level variables		
**Maternal Race**		
White	Ref	
Asian	1.30 (1.28, 1.32)	0.000
Black	1.73 (1.72, 1.75)	0.000
Latina	1.30 (1.28, 1.31)	0.000
**Maternal Nativity**		
US born	Ref	
Foreign born	0.85 (0.84, 0.86)	0.000
**Maternal Age**		
<=19	Ref	
20-24	0.91 (0.89, 0.93)	0.000
25-29	0.93 (0.91, 0.94)	0.000
30-34	1.05 (1.03, 1.06)	0.000
35-39	1.31 (1.29, 1.33)	0.000
40+	1.74 (1.70, 1.78)	0.000
**Maternal Education**		
Less than HS	Ref	
HS	0.88 (0.87, 0.89)	0.000
Some College/BA	0.74 (0.74, 0.75)	0.000
Graduate School	0.62 (0.61, 0.63)	0.000
**Delivery Payment**		
Private	Ref	
Public	1.18 (1.17, 1.19)	0.000
Self-Pay	1.03 (1.02, 1.05)	0.000
**Smoked in Pregnancy**		
No	Ref	
Yes	1.47 (1.45, 1.49)	0.000
**State-level Percent Foreign-Born**	1.00 (0.99, 1.00)	0.69
**State-level Percent Below FPL**	1.02 (1.01, 1.03)	0.000
**State-level Percent Republican**	1.00 (1.00, 1.01)	0.12

© 2021 Sudhinaraset M et al. *JAMA Network Open.*

eTable 8. Mixed Effects Logistic Models Estimating Odds of Preterm Birth and 2015 Immigrant Policies, Stratified by Nativity of Mother

	**Criminalizing Policy (range 0-6)**		**Inclusive Policy (range 0-14) P-value**	
	**AOR (95% CI)**	***P* value**	**AOR (95% CI)**	***P* value**
**US-born (N=2,653,654)**	1.02 (0.98, 1.01)	0.08	1.00 (0.98, 1.01)	0.87
**Foreign-born (N=801,860)**	1.02 (0.99, 1.05)	0.29	0.98 (0.96, 1.00)	0.03

Note: Models adjusted for both policies
Models adjusted for individual-level variables: maternal race, age, education, delivery payment type, and smoking status during pregnancy, and state level variables: state-level percent Republican voters in 2016 presidential election, and state-level percent living below the Federal Poverty Line

© 2021 Sudhinaraset M et al. *JAMA Network Open.*

eTable 9. Mixed Effects Logistic Models Estimating Odds of Preterm Birth and 2015 State Immigrant Policies, Stratified by Race and Nativity of Mother

	**Criminalizing Policy (range 0-6)**		**Inclusive Policy (range 0-14)**	
	**AOR (95% CI)**	***P* value**	**AOR (95% CI)**	***P* value**
**Asian (N=229,012)**				
US-born Asian (N=43,536)	0.95 (0.91, 1.00)	0.06	0.95 (0.93, 0.98)	0.000
Foreign-born Asian (N=185,476)	1.00 (0.95, 1.04)	0.83	0.98 (0.96, 1.01)	0.14
**Black (N=517,497)**				
US-born Black (N=429,754)	1.01 (0.98, 1.05)	0.45	1.00 (0.98, 1.02)	0.96
Foreign-born Black (N=87,743)	1.05 (1.00, 1.10)	0.03	0.98 (0.95, 1.00)	0.08
**Latina (N=852,180)**				
US-born Latina (N=448,854)	1.02 (0.99, 1.05)	0.30	1.00 (0.98, 1.02)	0.95
Foreign-born Latina (N=403,326)	1.02 (0.99, 1.06)	0.18	0.99 (0.97, 1.01)	0.23
**White (N=1,856,825)**				
US-born White (N=731,510)	1.02 (0.99, 1.05)	0.15	1.00 (0.98, 1.01)	0.78
Foreign-born White (N=125,315)	0.99 (0.95, 1.03)	0.68	0.97 (0.95, 0.99)	0.02

Note: Models adjusted for both policies
Models adjusted for individual level variables: maternal age, education, delivery payment type, and smoking status during pregnancy, and state-level variables: state-level percent foreign, state-level percent Republican voters in 2016 presidential election, and state-level percent living below the Federal Poverty Line

© 2021 Sudhinaraset M et al. *JAMA Network Open.*

eTable 10. Multilevel Logistic Models Estimating Odds of Preterm Birth, With Prenatal Care Removed From Inclusive Policy Index (N=3,455,514)

	**AOR (95% CI)**	***P* value**
State Immigrant Policy Context		
**Criminalizing Policy**	1.02 (1.00, 1.05)	0.09
**Inclusive Policy (without Prenatal Care)**	0.99 (0.97, 1.01)	0.39
**Prenatal Care**	1.00 (0.94, 1.07)	0.94
Individual-level variables		
**Maternal Race**		
White	Ref	
Asian	1.30 (1.28, 1.32)	0.000
Black	1.73 (1.72, 1.75)	0.000
Latina	1.30 (1.28, 1.31)	0.000
**Maternal Nativity**		
US born	Ref	
Foreign born	0.85 (0.84, 0.86)	0.000
**Maternal Age**		
<=19	Ref	
20-24	0.91 (0.89, 0.93)	0.000
25-29	0.93 (0.91, 0.94)	0.000
30-34	1.05 (1.03, 1.06)	0.000
35-39	1.31 (1.29, 1.33)	0.000
40+	1.74 (1.70, 1.78)	0.000
**Maternal Education**		
Less than HS	Ref	
HS	0.88 (0.87, 0.89)	0.000
Some College/BA	0.74 (0.74, 0.75)	0.000
Graduate School	0.62 (0.61, 0.63)	0.000
**Delivery Payment**		
Private	Ref	
Public	1.18 (1.17, 1.19)	0.000
Self-Pay	1.03 (1.02, 1.05)	0.000
**Smoked in Pregnancy**		
No	Ref	
Yes	1.47 (1.45, 1.49)	0.000
**State-level Percent Foreign-Born**	1.00 (0.99, 1.01)	0.77
**State-level Percent Below FPL**	1.02 (1.01, 1.03)	0.000
**State-level Percent Republican**	1.00 (1.00, 1.01)	0.13

© 2021 Sudhinaraset M et al. *JAMA Network Open.*

eTable 11. Mixed Effects Logistic Models Estimating Odds of Preterm Birth From Criminalizing and Inclusive Policy Variables, With Prenatal Care Removed From Inclusive Policy Index, Stratified by Nativity of Mother

	**Criminalizing Policy (range 0-6)**		**Inclusive Policy without Prenatal Care (range 0-13)**	
	**AOR (95% CI)**	***P* value**	**AOR (95% CI)**	***P* value**
**US-born (N=2,653,654)**	1.04 (1.01, 1.09)	0.03	0.99 (0.96, 1.01)	0.30
**Foreign-born (N=801,860)**	1.02 (0.99, 1.06)	0.18	0.98 (0.95, 1.00)	0.04

Note: Models adjusted for both policies
Models adjusted for individual-level variables: maternal race, age, education, delivery payment type, and smoking status during pregnancy, and state level variables: state-level percent Republican voters in 2016 presidential election, and state-level percent living below the Federal Poverty Line

© 2021 Sudhinaraset M et al. *JAMA Network Open.*

eTable 12. Mixed Effects Logistic Models Estimating Odds of Preterm Birth From Criminalizing and Inclusive Policy Variables, With Prenatal Care Removed From Inclusive Policy Index, Stratified by Race and Nativity of Mother

	**Criminalizing Policy (range 0-6)**		**Inclusive Policy Without PNC (range 0-13)**	
	**AOR (95% CI)**	***P* value**	**AOR (95% CI)**	***P* value**
**Asian (N=229,012)**				
US-born Asian (N=43,536)	0.96 (0.91, 1.00)	0.08	0.96 (0.92, 1.00)	0.06
Foreign-born Asian (N=185,476)	1.00 (0.96, 1.04)	0.87	0.99 (0.96, 1.02)	0.42
**Black (N=517,497)**				
US-born Black (N=429,754)	1.01 (0.98, 1.04)	0.52	0.99 (0.97, 1.01)	0.41
Foreign-born Black (N=87,743)	1.05 (1.00, 1.10)	0.03	0.98 (0.95, 1.01)	0.21
**Latina (N=852,180)**				
US-born Latina (N=448,854)	1.02 (0.99, 1.05)	0.28	1.00 (0.98, 1.03)	0.80
Foreign-born Latina (N=403,326)	1.02 (0.99, 1.06)	0.25	0.97 (0.95, 1.00)	0.05
**White (N=1,856,825)**				
US-born White (N=731,510)	1.02 (0.99, 1.05)	0.17	1.00 (0.98, 1.02)	0.73
Foreign-born White (N=125,315)	0.99 (0.95, 1.03)	0.65	0.96 (0.94, 0.99)	0.02

Note: Models adjusted for both policies
Models adjusted for individual level variables: maternal age, education, delivery payment type, and smoking status during pregnancy, and state-level variables: state-level percent foreign, state-level percent Republican voters in 2016 presidential election, and state-level percent living below the Federal Poverty Line

© 2021 Sudhinaraset M et al. *JAMA Network Open.*

eFigure 1. Predictive Margins with 95% CI, Criminalizing Policies

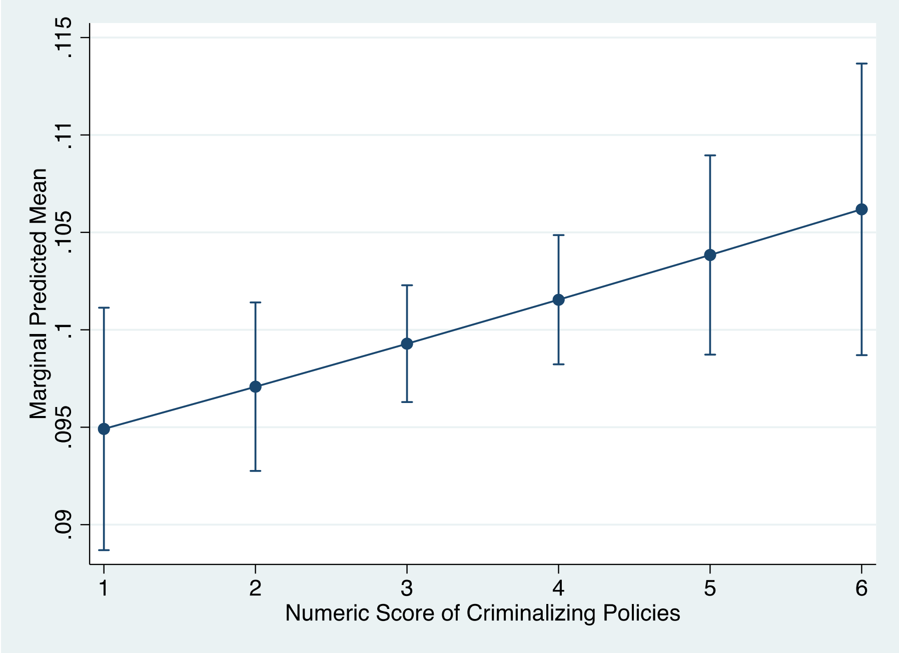

© 2021 Sudhinaraset M et al. *JAMA Network Open.*

eFigure 2. Predictive Margins with 95% CI, Inclusive Policies

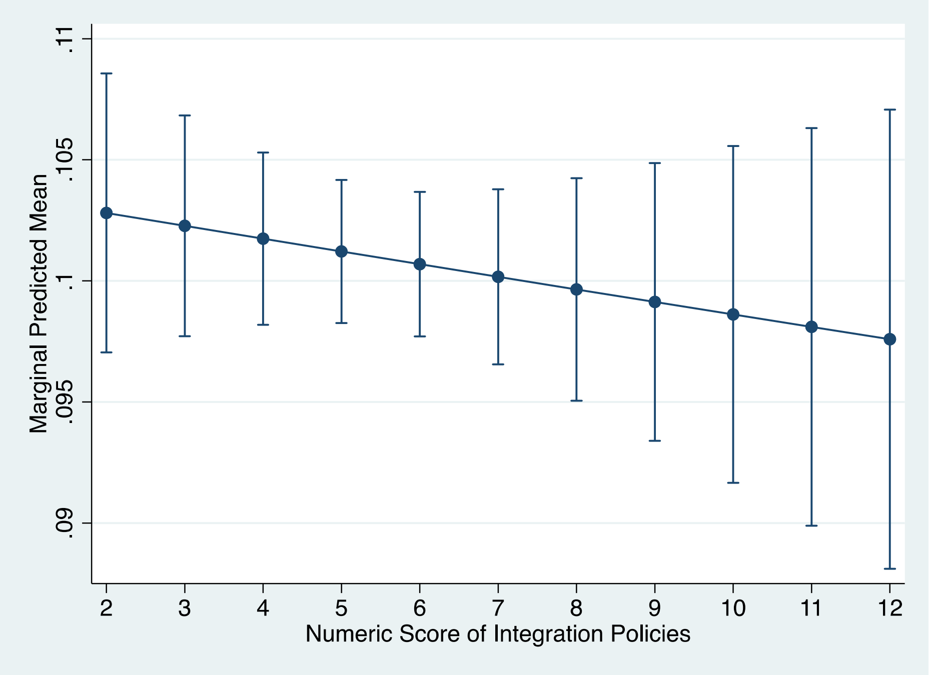

© 2021 Sudhinaraset M et al. *JAMA Network Open.*
